# Plasmonic Refractive Index Sensor Enhanced with Chitosan/Au Bilayer Thin Film for Dopamine Detection

**DOI:** 10.3390/bios12121124

**Published:** 2022-12-03

**Authors:** Faten Bashar Kamal Eddin, Yap Wing Fen, Josephine Ying Chyi Liew, Wan Mohd Ebtisyam Mustaqim Mohd Daniyal

**Affiliations:** 1 Department of Physics, Faculty of Science, Universiti Putra Malaysia, Serdang 43400, Selangor, Malaysia; 2 Functional Nanotechnology Devices Laboratory, Institute of Nanoscience and Nanotechnology, Universiti Putra Malaysia, Serdang 43400, Selangor, Malaysia

**Keywords:** surface plasmon resonance, refractive index sensing, neurotransmitters, polymer, optical sensor, light-matter interaction, performance enhancement

## Abstract

Surface plasmonic sensors have received considerable attention, found extensive applications, and outperformed conventional optical sensors. In this work, biopolymer chitosan (CS) was used to prepare the bilayer structure (CS/Au) of a plasmonic refractive index sensor for dopamine (DA) detection. The sensing characteristics of the developed plasmonic sensor were evaluated. Increasing DA concentrations significantly shifted the SPR dips. The sensor exhibited stability and a refractive index sensitivity of 8.850°/RIU in the linear range 0.1 nM to 1 µM with a detection limit of 0.007 nM and affinity constant of 1.383 × 10^8^ M^−1^. The refractive index and thickness of the CS/Au structure were measured simultaneously by fitting the obtained experimental findings to theoretical data based on Fresnel equations. The fitting yielded the refractive index values *n* (1.5350 ± 0.0001) and *k* (0.0150 ± 0.0001) for the CS layer contacting 0.1 nM of DA, and the thickness, *d* was (15.00 ± 0.01) nm. Then, both *n* and *d* values increased by increasing DA concentrations. In addition, the changes in the FTIR spectrum and the variations in sensor surface roughness and structure obtained by AFM analysis confirmed DA adsorption on the sensing layer. Based on these observations, CS/Au bilayer has enhanced the performance of this plasmonic sensor, which showed promising importance as a simple, low-cost, and reliable platform for DA sensing.

## 1. Introduction

Dopamine (DA), with the chemical formula 3,4-dihydroxyphenethylamine, is well known as one of the most essential neurotransmitters (NTs) in mammals’ central and peripheral neural systems since it conveys brain messages in the form of nerve impulses [1]. The normal concentration of DA in the human body affects the function of the central nervous system, supports blood pressure, and regulates physiological processes such as fine motor activity, stress, mental cognition, attention, inspiration, intuition, learning, motivation, emotions, and memory formation [2,3]. It also has a significant influence on the function of the hormonal, renal, and cardiovascular systems of the body. The physiological concentrations of DA vary in various body fluids. The concentration of DA in the blood is less than 130 pM, according to the Human Metabolome Database. Whereas its concentration in human cerebrospinal samples and urine is around 5 nM [4]. Considering the extensive physiological and pathophysiological impacts of DA, it is believed that abnormalities of DA concentrations in human blood and brain systems are associated with various diseases. DA deficiency is correlated with major neurological diseases such as Parkinson’s disease (PD) [5], Alzheimer’s disease [6], schizophrenia [7], and depression [8]. As a consequence, simple, sensitive, rapid, and accurate analytical methods to determine DA levels precisely would be useful for physiological studies and a critical marker for timely diagnostics and therapeutics. Up to now, huge efforts have been deployed, and many strategies have been used to detect DA in biofluids, such as high-performance liquid chromatography (HPLC) [9], capillary electrophoresis [10], mass spectroscopy [11], microdialysis techniques [12], Fourier transform infrared (FTIR) spectroscopy [13], flow injection [14], enzymatic approaches [15], electrochemical (EC) techniques [16] and other methods. Although these effective strategies are accurate and have their own features, they have drawbacks and limitations. Most of them take time, have limited sensitivity, need expensive equipment, and are not long-term stable. Furthermore, the sensor’s surface functionalization is challenging. These limitations prompted continued development to increase the sensitivity, selectivity, and biocompatibility of these sensors. Moreover, significant efforts have been made to develop optical-based techniques for detecting and quantifying DA at all physiological concentrations.

Today, surface plasmon resonance (SPR) based optical biosensor is considered among the most advanced technologies for studying biomolecular interactions [17]. This label-free sensor offers accurate and fast detection of biological and chemical analytes with good sensitivity. It has proven its potential for application in different important fields, such as clinical and medical diagnostics [18,19], food-safety analysis [20], environmental protection [21,22,23,24], and others. The ability to tune the optical characteristics of the plasmonic-based sensors throughout a broad frequency range, from visible to infrared, enables in vivo applications of these sensors, getting the benefit of the transparency of the blood and tissues in the near-infrared range [25]. The latest developments in plasmonic and metamaterial-based plasmonic biosensors for virus detection were discussed by Hassan et al. [26]. The detection limits reported for the developed SPR sensors were extremely low. Among the effective reported sensors, the simple planar SPR biosensor showed the potential to detect the HIV virus successfully down to 48 fM. Surface-enhanced spectroscopic methods, such as surface-enhanced Raman spectroscopy (SERS) and surface-enhanced infrared absorption (SEIRA), have demonstrated their effectiveness in sensing specific biological and chemical molecules. These techniques are thought to be complementary to refractive index sensors. The high electric field intensities of nanostructured surfaces influence the sensitivity of these techniques resulting in signal enhancement factors on the order of single-molecule spectroscopy utilizing SERS substrates and zeptomoles for the SEIRA method [27,28]. Also, the whispering gallery mode (WGM) laser emission’s high intensity makes it very sensitive to tiny variations at the resonator interface, such as changes in the effective refractive index or the cavity’s size and shape. These variations cause detectable shifts in the resonance frequency, which may be used to design reliable spectroscopic encoding methods for identifying molecular interactions in biological samples or to observe changes in environmental circumstances. Capocefalo and co-workers conducted optical sensing of the microresonators by progressively doping the liquid gain medium with compounds of varying refractive index and random distribution. When titanium-dioxide micropowder (TiO_2_) was used separately as a dielectric component, they observed the lowest detection limit of 16 µg/mL [29]. Furthermore, the WGM optical testing kit demonstrated remarkable sensitivity in identifying particular immunoglobulin antibodies. The essential component of the kit developed by Yue and co-workers was a self-assembled silica microsphere decorated with the SARS-CoV-2 virus’s nucleocapsid proteins (N-proteins). The detection limits for N-protein antibody immunoglobulin (N-IgG) and immunoglobulin M (N-IgM) were determined to be 1 µg/mL and 2 µg/mL, respectively [30]. In addition, photonic crystals composed of alternating regions of high and low dielectric constants with 1D, 2D, or 3D periodic array arrangements have been used as sensors due to their well-defined physical properties and the presence of energy bands [31,32,33,34]. The photonic crystals’ sharp resonant optical response makes it considerably simpler to detect minor variations in the reflectivity spectrum, resulting in detection limits on the femtomolar concentration.

Over the last two decades, SPR spectroscopy has received extensive attention as a powerful analytical technique and has been developed in different configurations to detect a diversity of analytes. This plasmonic-based technique is highly sensitive to boundary conditions. This made it a refractometer that can detect and quantify even slight variations in the sensing medium’s refractive index induced by molecule adsorption on the sensing film. When the refractive index changes owing to the target’s strong binding to the sensor film, the SPR response varies where the angular or spectral position of the SPR dip shifts to represent specific features of the system and offer details about the kinetics of the adsorption process on the sensor surface [35,36,37,38]. Yet, detecting DA using SPR sensors is still in its early phases, even though the preliminary results are encouraging [4,39,40,41,42,43,44,45,46,47,48,49,50,51]. Because of their significant benefits of high specificity and sensitivity, cheap cost, sensing capabilities in real-time, no labeling required, and ease of preparation, SPR sensors have demonstrated their effectiveness as superior to other diagnostic tools. However, detecting targets of extremely low concentrations using an SPR-based sensor requires increasing the sensor’s sensitivity.

Chitosan (CS) is a nontoxic biopolymer found abundantly in nature. CS is derived from chitin, a natural organic molecule that may be taken from the exoskeletons of crustaceans and insects. This polymer has a lot of amino (–NH_2_) and hydroxyl (–OH) functional groups [52]. CS biopolymer and its derivatives were selectively utilized to stabilize plasmonic NPs, semiconductor NPs, luminescent NPs, and photoluminescent complexes in the field of optically active materials [53]. Therefore, in this work, CS thin films were developed. Then, these films were incorporated into the SPR technique for DA detection. The performance of this plasmonic sensor was evaluated. In addition, the structural and optical characteristics of the sensor film were investigated before and after interaction with DA. 

## 2. Materials and Methods

### 2.1. Chemical Preparation

Chitosan with medium molecular weight (MMW) (CS, 75–85% deacetylated, MW = 90,000–310,000 Da), acetic acid (assay ≥ 99.7%), and dopamine hydrochloride (DA, white powder, MW = 189.64 g/mol) were obtained from Sigma-Aldrich (St. Louis, MO, USA). To get a homogenous CS solution, 0.4 g of MMW CS was added to 50 mL of 1% acetic acid and dissolved by stirring at room temperature for 24 h [54]. A DA aqueous solution with a concentration of 1 M was produced by dissolving 9.482 g of DA in 50 mL of deionized water (DW). Then, DW was utilized to dilute the DA solution based on this dilution formula (*M*_1_*V*_1_ *= M*_2_*V*_2_) to produce extremely low concentrations.

### 2.2. Preparation Process of Sensor Chip 

**Figure 1:**
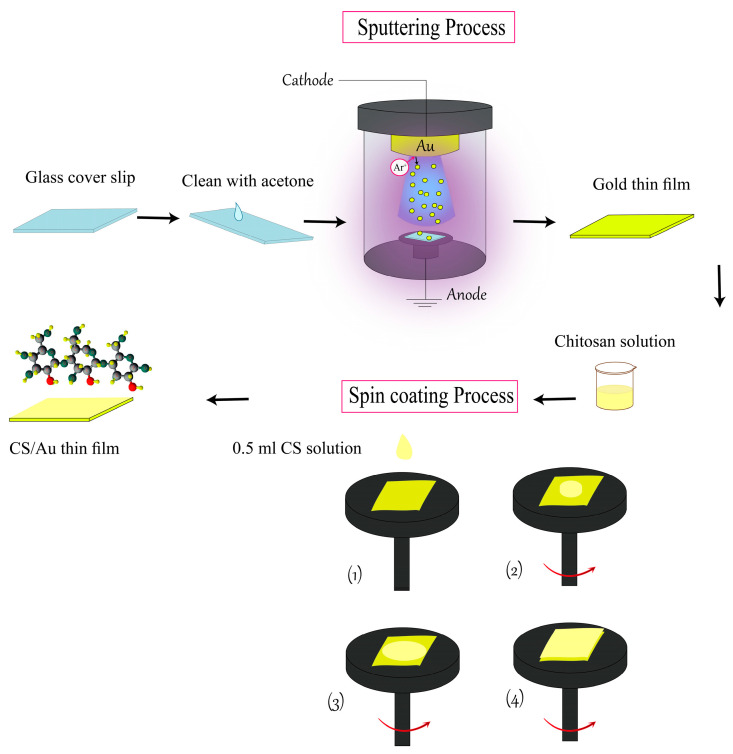
Process of sensor chip preparation.

Glass coverslips of 24 × 24 mm and thickness of 0.13–0.16 mm were obtained from Menzel-Glaser. Acetone was utilized for cleaning the coverslips sufficiently to remove the dust and fingerprint available on their surfaces, ensuring that no surface contamination or residual adsorbents were present to impact the accurate experimental measurements and sensor performance. Acetone was chosen since it is volatile, as well as it does not react with the coverslips. Then the glass slip was coated with a gold thin layer using a K575X sputter coater (Quorum Technologies, West Sussex, UK). The sputtering process was run at a current of 20 mA and a voltage of 2.2 kV and lasted 67 s. Then, 0.5 mL of CS solution was applied uniformly to the surface of the gold thin layer on the glass substrate, and spin coater (P-6708D) was employed for 30 s to coat CS sensing layers on the top of the gold thin films (CS/Au) at 2000 rpm as shown in Figure 1. 

### 2.3. Sensing System Configuration

**Figure 2:**
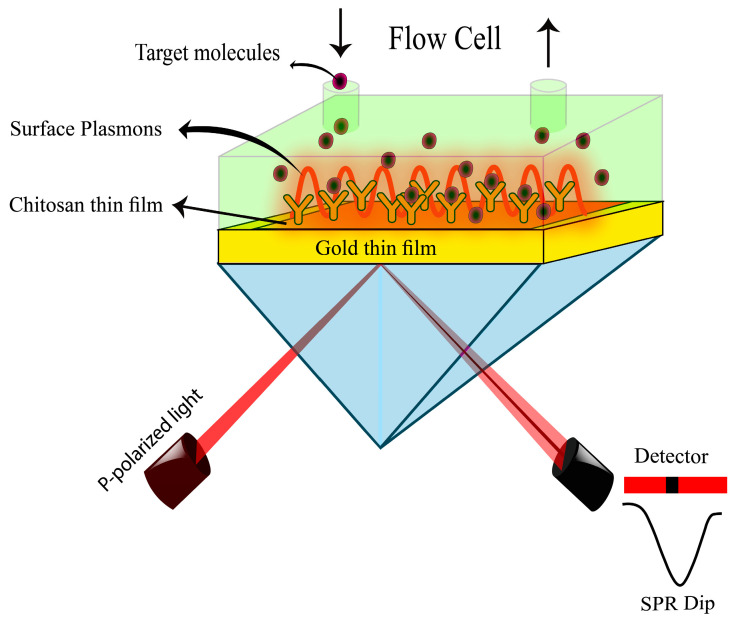
SPR configuration.

The sensing characteristics of CS/Au thin film in contact with DA solution were investigated and analyzed using a home-built SPR spectroscopy in Kretschmann configuration based on angular interrogation. It consisted of a He-Ne laser which was used as an excitation beam with a fixed wavelength at 632.8 nm, a beam chopper, a linear polarizer, a tiny pinhole, a triangular prism with a refractive index of 1.77861, an optical programmable gyratory platform driven by a motion controller (Newport model MM 3000) with a resolution of 0.001°, a photodetector, and a lock-in amplifier, as shown in Figure 2. The uncoated surface of the SPR chip was linked to the prism using the index matching liquid, and the flow cell that contains the sample solution made touch with the sensor chip’s surface [55,56,57,58]. Afterward, SPR tests were performed in a dark environment using CS/Au sensor films in contact with DW initially, then with different concentrations of DA. To get the reference signal, DW was injected into the attached flow cell and contacted the sensing layer. Following that, DA solutions with different concentrations were sequentially injected into the cell to conduct the experiments and quantify the intensity of the reflected light as a function of the incidence angle. After the angular spectral analysis and the evaluation of the ability of CS/Au bilayer film to sense low concentrations of DA based on the variations in the medium’s refractive index, the experimentally collected data were processed to obtain the optical characteristics of DA solution, gold thin film, and sensor film, which were evident and quantifiable after fitting experimental data to theoretical data.

### 2.4. Structural Characterization Techniques 

FTIR spectra of CS/Au bilayer films prior to and after interactions with DA were recorded in an ambient atmosphere at room temperature with ALPHA II FTIR Spectrometer plus Platinum ATR Accessory in the range 400–4000 cm^−1^ with a spectral resolution of 1 cm^−1^. The surface morphology images and the roughness changes analysis of sensor films before and after introducing DA solution were observed at room temperature using Bruker Dimension Edge AFM in PeakForce Tapping mode with a scanning size of 5 μm × 5 μm. The cantilever spring constant is 0.4 N/m, and its dimensions are T (650 nm), L (115 µm), and W (25 µm). The radius of curvature of the AFM tip is <10 nm. The front side of the cantilever is without coating, while its back side is coated with reflective Al.

## 3. Results and Discussion

### 3.1. Structural Characterization

**Figure 3:**
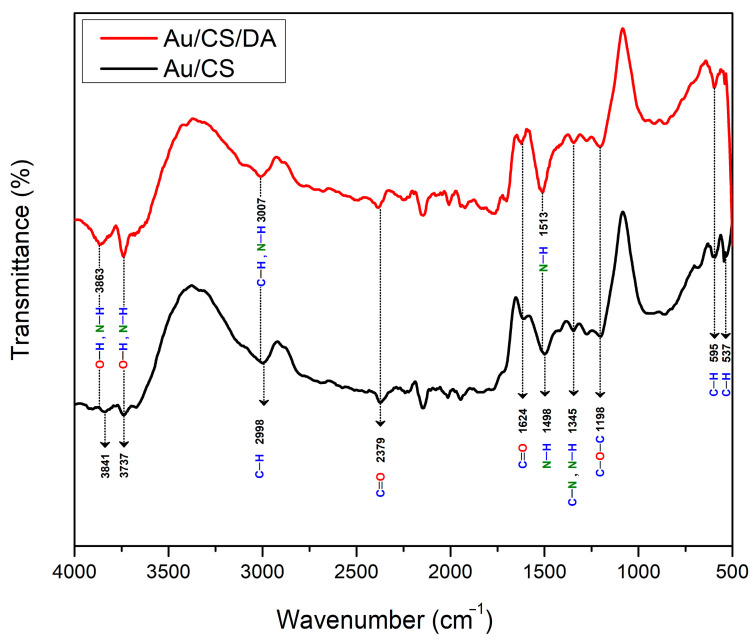
FTIR spectra of CS/Au bilayer film prior to and after interaction with DA.

FTIR spectra recorded for CS/Au bilayer film prior to and after adsorption of DA on its surface are shown in Figure 3. The appeared peaks at 3841 and 3737 cm^−1^ are ascribed to the stretching vibrations of the O–H and N–H bonds [59,60]. The peaks located at 2998 cm^−1^ are attributed to C–H stretching [61,62], and the peaks located around 2379 and 1624 cm^−1^ originated due to the stretching vibrations of C=O [63,64]. The peak available at 1498 cm^−1^ is due to the stretching vibration band of N–H, and the peak at 1345 cm^−1^ is related to the vibrations of C–N and N–H bonds [62,65]. The peak found at 1198 cm^−1^ is corresponded to C–O–C stretching [64]. The peaks available at 595 and 537 cm^−1^ are linked to C–H bending [66]. After introducing DA to be in contact with the sensor film, the peak at 3841 cm^−1^ was shifted to 3863 cm^−1^ with higher intensity and became wider due to the overlapping with the N–H stretching vibrations. Also, the peak at 3737 cm^−1^ originating from O–H and N–H vibrations increased in intensity. Further, the C–H band at 2998 cm^−1^ was shifted to 3007 cm^−1^ due to vibrations of the N–H bond of DA. Also, N–H band at 1498 cm^−1^ was replaced at 1513 cm^−1^ and became more intense. Furthermore, the C–H band at 537 cm^−1^ has diminished. These results indicate that the structure of the sensor film was changed due to DA binding to its surface. 

**Figure 4:**
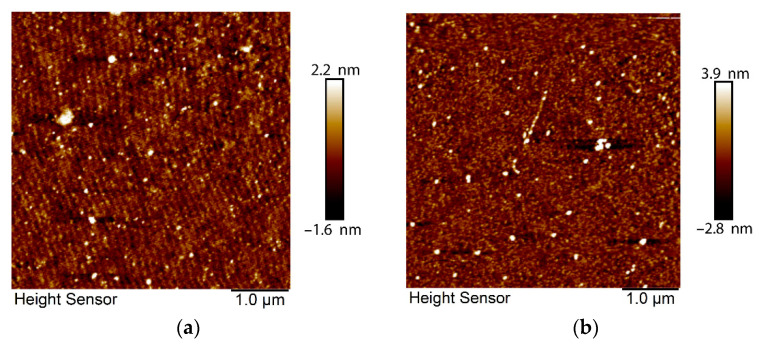

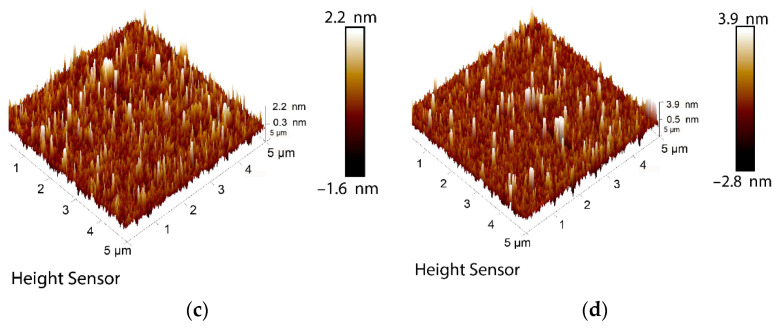
AFM images of CS/Au bilayer film: (**a**) 2D image before interaction with DA; (**b**) 2D image after interaction with DA; (**c**) 3D image before interaction with DA; and (**d**) 3D image after interaction with DA.

Structural characterization of the sensor film was continued using AFM with a scanning size of 5 μm × 5 μm. Figure 4 illustrates the surface morphology of CS thin film prior to (Figure 4a) and after (Figure 4b) interaction with DA. Many sharp peaks were apparent in the 3D image of CS before exposure to DA (Figure 4c). When DA was introduced, these peaks decreased, and they became wider and longer (Figure 4d). The average roughness of the sensor surface *Ra* was increased from 0.336 nm to 0.552 nm, and the standard deviation of the Z values *Rq*, also known as the RMS roughness (root mean square), was increased from 0.514 nm to 0.979 nm after DA injection. These changes in the sensor surface roughness and structure confirm DA adsorption on the sensing layer. The sensor structure maintained its stability during the experiments, where the angular shift of SPR dips increased with increasing DA concentrations. This was further validated by AFM images, which did not indicate degradation of the sensor surface after several measurements.

### 3.2. SPR Reflectivity Curves for DA on CS/Au Bilayer Film

**Figure 5:**
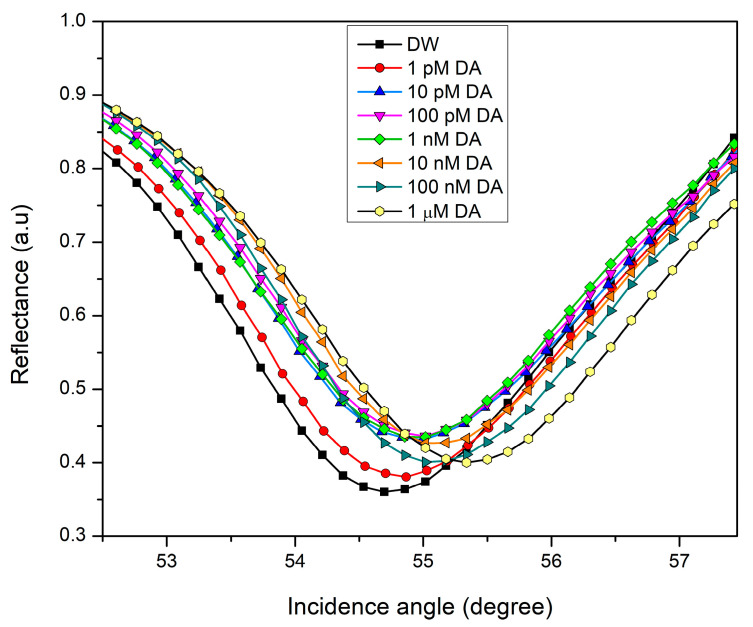
The experimental SPR reflectivity curves of CS/Au sensor film exposed to different concentrations of DA.

The measurements were conducted using DW and DA solutions of various concentrations ranging from 0.001 nM to 1000 nM. First, CS/Au bilayer film made contact with DW to record the reference signal and determine the resonance angle that corresponds to the minimum value of the reflectivity. Continuing the measurements, the resonance angles determined using SPR reflectivity curves for 0.001, 0.01, and 0.1 nM of DA were not shifted from the SPR angle obtained using DW. After increasing the DA concentration to 1 nM, the SPR dip was moved to the right with an angular shift Δ*θ* calculated by subtracting the resonance angle obtained for DW from the resonance angle obtained for each concentration of DA. For 10 nM DA, the resonance occurred at a higher angle. As shown in Table 1, the continuous increase in the concentration of DA solution that contacted the sensor caused the resonance to happen at greater angles. Figure 5 depicts SPR spectra with considerable shifts of angular resonance position as DA concentration increases.

### 3.3. Sensing Characteristics Analysis

The limit of detection (LOD) of the SPR sensor denotes the lowest concentration of the analyte detectable by the sensor, where the sensor response for this sample concentration differs from its baseline response [67,68]. Furthermore, the International Union of Pure and Applied Chemistry (IUPAC) defines the LOD as follows [69]:

(1) LOD=m σ

where *m* is a numerical factor chosen according to the confidence level desired (typically 3), and *σ* is the standard deviation of the blank measures. Using this CS/Au bilayered structure, the detection limit was calculated based on the IUPAC definition to be 0.007 nM which is lower than other sensors that used CS in DA sensing. SERS sensor developed by Kim and Kang using CS-Au nanoshell showed a distinct change in the intensity of Raman scattering across the DA concentration range (1 mM–10 mM) [70]. Huang et al. [71] developed an electrochemical sensor and modified the glassy carbon electrode (GCE) with Au, CS, and carbon dots (CDs) to detect DA. In 0.2 mM DA solution, they investigated the electrochemical responses of DA at different electrodes (CS/GCE, CDs-CS/GCE, and Au/CDs-CS/GCE). Their findings revealed that when CS was cast on the GCE, the redox peaks vanished. However, when CDs-CS or Au-CS were placed on the electrode’s surface, the redox peaks grew somewhat. The redox peaks increased significantly only for Au/CDs-CS/GCE, where DA was detected down to 0.001 μM in a linear range of 0.01–100.0 μM. Additionally, Wang et al. [72] reported the cyclic voltammograms of 1 mM DA was recorded on CS/GCE and other electrodes. The clear voltammetric peaks of DA oxidation on both CS/GCE and GCE were not observed. They were detected for the electrode that had been treated using a graphene-CS composite. 

**Figure 6:**
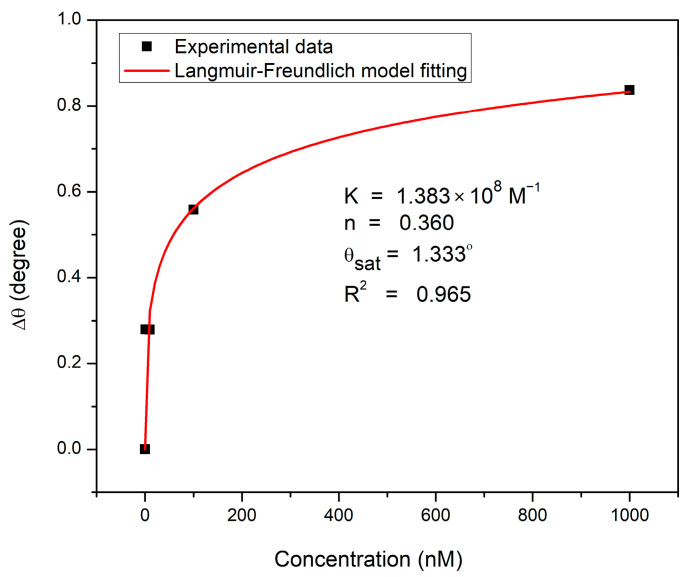
Experimental and fitted data to Langmuir and Freundlich model for DA adsorption on CS/Au sensor film.

The binding affinity of the CS/Au-based sensor towards DA molecules was investigated by the non-linear fitting of the experimental data based on the Langmuir and Freundlich isotherm model, as shown in Figure 6. This model merges the Langmuir model and Freundlich model to provide details on the heterogeneity in the adsorption behavior throughout a wide range of concentrations up to saturation, transcending the Freundlich model’s restriction that arises with analytes of high concentrations. Therefore, the Langmuir and Freundlich model will be reduced to the Freundlich model for analytes with low concentrations. However, at high concentrations, it predicts monolayer adsorption and indicates the Langmuir model. The Equation associated with the Langmuir and Freundlich model is stated as follows [73]:

(2) $$\Delta \theta =\frac{\Delta \theta_{max}\;K\;C^{n}}{1+KC^{n}}$$

where $\Delta \theta_{max}$ denotes the maximum angular shift of SPR dip, *K* denotes the Langmuir and Freundlich affinity constant, *C* denotes the analyte concentration, and n indicates the system heterogeneity index. The obtained correlation coefficient (R^2^ of 0.965) proved that the Langmuir and Freundlich isotherm model suited the experimental findings well with an affinity constant of 1.383 × 10^8^ M^−1^. The Δ*θ_max_* value produced by this model at saturation was higher than the experimental value (0.837°), and the Langmuir and Freundlich exponent value was 0.360. This low value of n reflects the low intensity of adsorption.

The angular width of the SPR curve at half the maximum reflectance is termed the full-width half maximum (FWHM) [74,75,76]. The FWHM value is critical for determining sensor accuracy. The detection accuracy is inversely proportional to the FWHM of the SPR curve; hence the FWHM value should be as minimal as possible to minimize the error in determining the resonance angle [77]. The Gaussian fitting procedure was performed for all obtained SPR curves to determine their FWHM values. The FWHM calculated for the SPR curve when DW contacted the sensor surface was 2.869°, and the detection accuracy was 0.349 (deg^−1^). The measurements performed for DA produced SPR reflectance curves that were a little wider than the SPR curve for DW, where the FWHM value determined for 1 nM DA was 2.923°.

Another important parameter for analyzing the sensor performance and quantifying its precision is the signal-to-noise ratio (SNR) which can be calculated as stated below [78]:

(3) $$\mathrm{SNR}=\frac{\Delta \theta }{\mathrm{FWHM}}=\Delta \theta \times \;\mathrm{Detection}\;\mathrm{accuracy}$$

**Figure 7:**
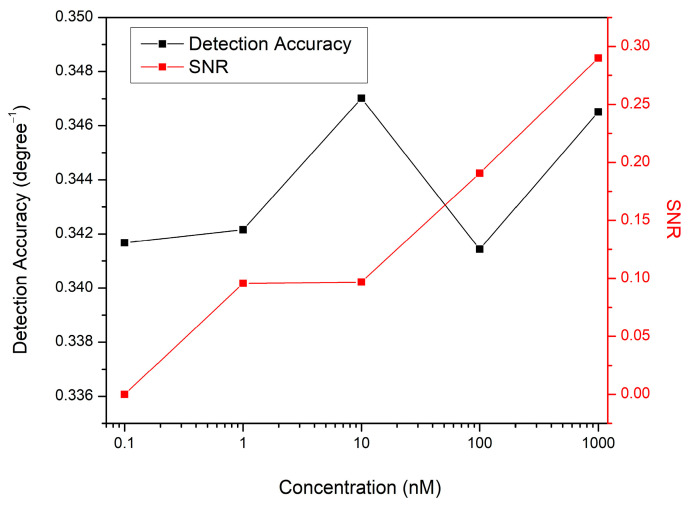
Detection accuracy and SNR variations with DA concentrations.

Figure 7 shows how SNR and detection accuracy change as a function of DA concentration. Notably, raising the DA concentration reduced the noise in the SPR signals, which enhanced the SNR values for the suggested sensor. This is because increasing the concentration of DA solution increased the resonance angular shift, which has a higher influence than detection accuracy in estimating the SNR value.

### 3.4. Optical Characterization and Refractive Index Sensitivity of CS/Au-Based Sensor

**Figure 8:**
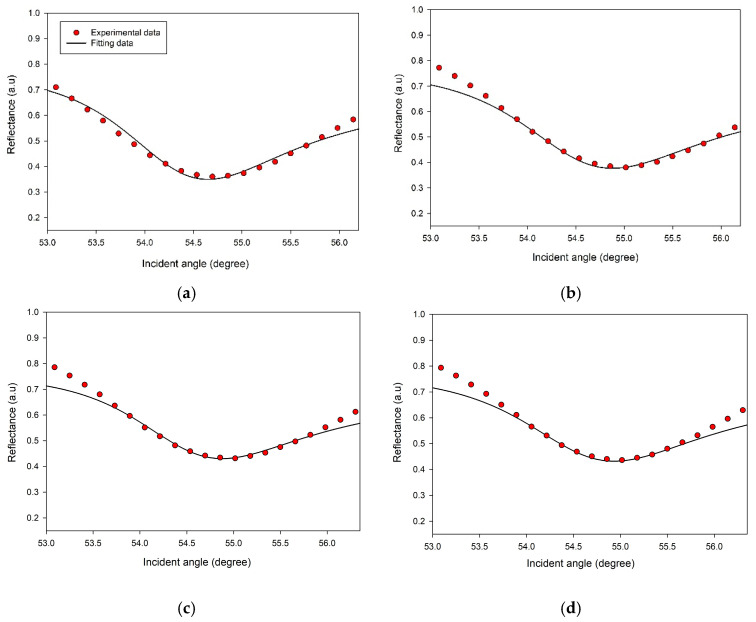

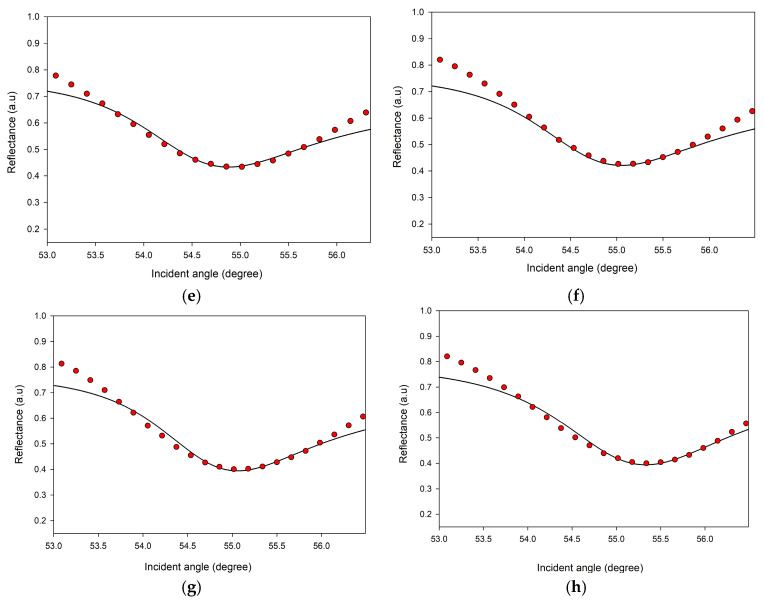
The experimental and fitted reflectance curves related to CS/Au bilayer film subjected to different concentrations of DA: (**a**) 0 pM; (**b**) 1 pM; (**c**) 10 pM; (**d**) 100 pM; (**e**) 1 nM; (**f**) 10 nM; (**g**) 100 nM; (**h**) 1 µM.

The different characteristics of the proposed SPR sensor with multilayered (i.e., substrate–Au layer–CS layer–DA solution) structure were studied using Fresnel Equations [79]. The simulation was run based on the transfer matrix method (TMM) in a MATLAB environment. By fitting the experimental reflectance curves for the used gold film and DA solutions to theoretical data, the gold layer’s refractive index values, *n* and *k,* were (0.2650 ± 0.0001) and (3.8590 ± 0.0001), respectively, and its thickness *d* was (59.60 ± 0.01) nm. From the previous work, the fitting of experimental curves for the gold layer subjected to DA yielded refractive index values for DA solution with concentrations less than 10 pM, which was the same as DW [51]. For higher concentrations of DA up to 100 nM, the *k* value was found to be (0.0030 ± 0.0001). However, the DA sample of 1 µM had the same *n* (1.3333 ± 0.0001), but the *k* value was (0.0050 ± 0.0001). After fitting the experimental reflectance curves of CS thin films based on these findings, as depicted in Figure 8, the CS layer’s refractive index values *n* and *k* were (1.5350 ± 0.0001) and (0.0150 ± 0.0001) respectively, and the film thickness was (15.00 ± 0.01) nm. Using 1 pM, 10 pM, and 100 pM of DA yielded the same *n*, *k*, and *d* values of CS film obtained using DW, and no SPR shift was observed. The fitting findings showed that the *n* values of the CS layer increased to 1.6300 as the DA concentration was changed from 0 µM to 1 µM, while the *k* values decreased to 0.0110. The thickness of this active layer increased by 1.25 nm during the detection of DA with these low concentrations, as seen in Table 2. The variations of the imaginary part of the refractive index of the gold film, also known as the damping coefficient, was investigated where the SPR reflectance curve for CS/Au structure exposed to DW was fitted to theoretical data. The decrease of the damping coefficient from 3.8590 to 3.7590 reduced the absorption coefficient α, increasing the transmittance. When the damping coefficient was increased above 3.8590, the reverse occurred. Where the damping coefficient is proportional to the absorption coefficient as follows [55]:

(4) $$k=\frac{\alpha \;\lambda }{4\pi }$$

where λ is the wavelength of the laser used. 

The changed values of the damping coefficient of the gold layer have changed the resonance properties and induced the resonance to happen at higher incidence angles. 

Given the importance of sensitivity during the evaluation of the sensor’s performance, high sensitivity is always desirable. The refractive index sensitivity of this SPR sensor was also evaluated. It is defined in the angular interrogation mode as the change in Δ*θ* per the change in the real part of the sensor film’s refractive index and represented as follows [80,81]:

(5) $$S_{RI}=\frac{\Delta \theta }{\Delta n}$$

**Figure 9:**
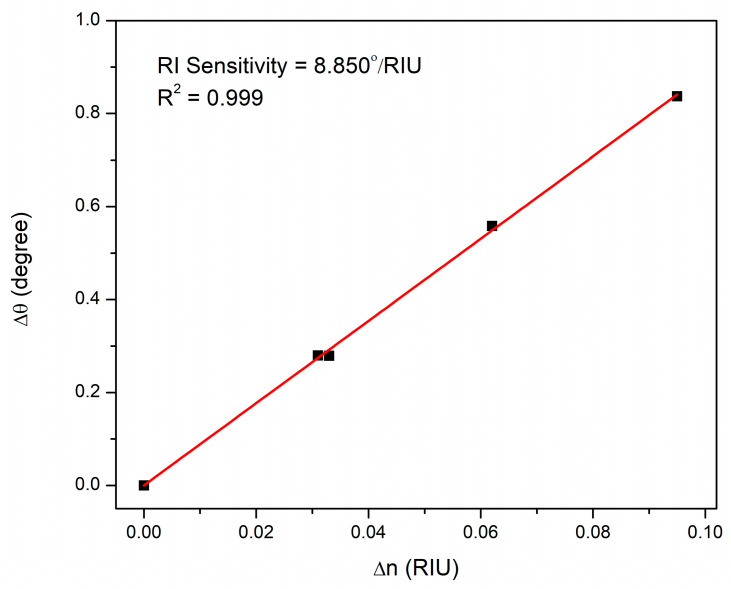
Linear fitting of the change in resonance angle versus change in CS/Au sensor film’s refractive index exposed to different concentrations of DA.

Figure 9 shows the linear fitting of the calculated values from experiments (Δ*θ*) and theoretical fitting (Δ*n*) stated in Table 2. This CS-based SPR sensor has a refractive index sensitivity of 8.850°/RIU based on the slope of the fitting line with an R^2^ value of 0.999.

As far as we are concerned, the preliminary results of the previously developed DA SPR sensors are encouraging. Nevertheless, their reports on the sensing performance are very limited. Therefore, the sensing performance of the proposed sensor has been evaluated in terms of sensitivity, binding affinity, LOD, and accuracy. Manaf et al. [45] developed a four-layer coating structure using SU8 waveguide, platinum, platinum nanoparticles, and plastic. Their SPR sensor had the potential to detect DA down to 0.05 nM, while the fiber optic SPR sensor developed by Sharma and Gupta using molecular imprinted graphene nanoplatelets/tin oxide (SnO_2_) nanocomposite exhibited good selectivity to DA, and the reported detection limit was 0.031 µM [46]. Cao and Mcdermott proposed an SPR sensor using a complex procedure to detect DA down to 200 fM with a binding affinity of (3.1 ± 1.4 nM) [4]. In this work, the biopolymer-based SPR sensor was designed easily to detect DA with an affinity constant of 1.383 × 10^8^ M^−1^. Moreover, the reported studies did not investigate DA binding behavior on the sensor surface using structural measurements. In addition, the characterization of the refractive index of DA and the sensor film, as well as the determination of the sensor film thickness, were reported only by Manaf et al. [45]. Therefore, the characterization of the structural and optical properties of this sensor film and the determination of its thickness before and after interactions with DA were done in this work. Although the previously developed sensor based on CS-GQDs nanocomposite film could detect DA of extremely low concentrations [49], this SPR sensor based on CS/Au bilayer film provides the advantages of cost-effectiveness and ease of preparation [82]. Furthermore, this biopolymer is an eco-friendly material abundantly available [83]. This proposed sensor was compared to other sensors that used the same material without and with additional materials in terms of the lowest detection limits of DA, as shown in Table 3. 

It is obvious that the suggested sensor outperformed the SERS sensor based only on CS, which had a detection limit of 1 mM and other electrochemical sensors that used CS with CDs, graphene, graphene quantum dots, and reduced graphene oxide.

## 4. Conclusions 

In this work, a plasmonic sensor was constructed employing a CS/Au bilayered structure for DA detection, and the performance of the developed sensor was successfully examined. This simple and low-cost sensor showed good stability and a sensitivity of 8.850°/RIU with an LOD of 0.007 nM and a binding affinity constant of 1.383 × 10^8^ M^−1^. The experimental results revealed increased resonance angular shift by increasing DA concentrations. The fitting of the experimental findings to theoretical data demonstrated the capability of this sensor to detect small variations in the refractive index and the thickness of the sensing structure simultaneously when DA solutions were introduced into the system at progressively increasing quantities. Furthermore, FTIR and AFM analysis of the sensor film structure validated the binding and attachment of DA on the surface of CS/Au film. The obtained results can be regarded as a first step toward the practical application of this biopolymer-based plasmonic sensor. More studies are important to evaluate the sensor selectivity in the presence of other interferences, such as epinephrine, serotonin, and uric acid, found in actual biological samples.

## Figures and Tables

**Table 1:** The SPR angle and angular shift of the SPR dip for a CS/Au bilayer structure exposed to varied DA concentrations.

DA Concentration (nM)	Resonance Angle (deg)	Δ*θ* (deg)
0.000	54.675	0.000
0.001	54.675	0.000
0.01	54.675	0.000
0.1	54.675	0.000
1	54.955	0.280
10	54.954	0.279
100	55.234	0.559
1000	55.512	0.837

**Table 2:** Refractive index of DA and CS film, thickness values for CS film, change of the real part of CS film’s refractive index Δ*n*, and change of resonance angle Δ*θ*.

DA Concentration (nM)	Refractive Index of DA Solution		Refractive Index of CS Layer Exposed to DA		Thickness of CS Layer *d* (nm) (±0.01)	Δ*n* (±0.0001)	Δ*θ*
	Real Part, *n* (±0.0001)	Imaginary Part, *k* (±0.0001)	Real Part, *n* (±0.0001)	Imaginary Part, *k* (±0.0001)			
0	1.3333	0.0000	1.5350	0.0150	15.00	0.0000	0.000 ± 0.001
0.001	1.3333	0.0000	1.5350	0.0150	15.00	0.0000	0.000 ± 0.001
0.01	1.3333	0.0030	1.5350	0.0150	15.00	0.0000	0.000 ± 0.001
0.1	1.3333	0.0030	1.5350	0.0150	15.00	0.0000	0.000 ± 0.001
1	1.3333	0.0030	1.5660	0.0130	15.50	0.0310	0.280 ± 0.018
10	1.3333	0.0030	1.5680	0.0130	15.50	0.0330	0.279 ± 0.017
100	1.3333	0.0030	1.5970	0.0110	15.90	0.0620	0.559 ± 0.022
1000	1.3333	0.0050	1.6300	0.0100	16.25	0.0950	0.837 ± 0.021

**Table 3:** Comparison of the proposed SPR sensor with different CS-based sensors for DA detection.

Materials	Technique	LOD	Reference
Au/CS	SERS	1 mM	[70]
Au@CDs/CS	Electrochemical	0.001 µM	[71]
Graphene/CS	Electrochemical	5 µM	[72]
CS-CDs/GCE	Electrochemical	11.2 nM	[84]
Nitrogen-doped graphene quantum dots-CS	Electrochemical	0.145 µM	[85]
Reduced graphene oxide-Au nanoparticles-CS/silica	Electrochemical	0.3 µM	[86]
CS /Au	SPR	0.007 nM	This work

## Data Availability

Not applicable.
